# Miscellaneous

**Published:** 1896-02

**Authors:** 


### MISCELLANEOUS.

### ELECTROLYSIS FOR THE SURGICAL TREATMENT OF STRICTURES.*

It affords me great pleasure to have the honor of being allowed through the kindness of your president to present to you a new instrument which I have devised and called "electrolyser," for the surgical treatment of strictures by the "linear electrolysis" method.

It is a well known fact that electrolysis has been discarded on account of the imperfect instruments which were used. My electrolyser has all the advantages of the urethrotome and none of its inconveniences. It looks like a small whip of which the handle contains a metallic wire projecting from the end which connects with the flexible part. This instrument, being first introduced into the urethra, is connected with the negative pole of a continuous current battery, and the positive pole is connected near the affected part, on the front of the thigh or over the pubes; then the current is turned on.

The operation which is almost painless, requires thirty seconds (on an average), with a current of strength of at least ten milliampères, as indicated by means of a galvanometer. The electrolyser remains perfectly cool during the operation. In nearly all cases there is no bleeding, or but very little. The urethra is made aseptic before and after the operation, in order to prevent fever. I never allow a sound to remain permanently in the urethra for any length of time after the operation.

---

*By J. M. Fort, M.D., Professor of Anatomy in Éthe cole Pratique of the Paris Faculté de Médecine. Read before the Section in Genito-urinary Surgery of the New York Academy of Medicine, Tuesday, November 12, 1895.

Usually the wound resulting from electrolysis heals quickly without any local treatment whatever, and often the patient can attend to business immediately after the operation.† In nearly all cases I pass a sound the third day after the operation, also the day after. I instruct the patient to pass a sound, No. 22 or No. 24 F., every month and every other month.

With the urethrotome, which cuts blindly, the surgeon cannot ascertain the degree of density of the tissue of a stricture. On the contrary, by means of electrolysis, which merely produces a molecular destruction of the stricture, although the instrument remains cool, I have been able to demonstrate that there are two classes of strictures—"soft and hard." Hard strictures are in the proportion of one against five soft ones.

The time required to perform the operation varies with the density of the stricture. Some strictures are so hard that they cannot be successfully operated upon by electrolysis.

If my American colleagues who are familiar with the French language are willing to refer to one of my books entitled *Traitement des rétrécissements par l'électrolysis linéaire* (this book can be procured at the library of the Academy of Medicine), they may find it quite interesting, as it will enable them to understand the improvements which have gradually been introduced in the applications of electrolysis to surgery during the last fifteen years. They will also understand how I have applied electrolysis to the treatment of strictures of the urethra, uterus, rectum, and esophagus.

Up to date, I have performed in Europe a hundred and thirty-five operations on strictures of the esophagus (recorded in my book), and with the exception of those which were caused by malignant growths of the wall of the esophagus all recovered.

It has been my good fortune to meet here some leading surgeons who are authorities in the treatment of strictures, and I am very grateful to them for their kindness in giving me the opportunity to demonstrate the advantages of my method in operating upon some of their patients.—*N. Y. Medical Journal,* Nov. 6, 1895.

---

†When the wound does not heal, I merely prescribe injections morning and evening with one part of hydrozone to twenty parts of water.

